# Lacrimal Sac Cysticercosis: A Rare Site for Manifestation

**DOI:** 10.1155/2014/961815

**Published:** 2014-11-13

**Authors:** Amita Raoot

**Affiliations:** University College of Medical Sciences, Delhi University, India

## Abstract

*Cysticercosis*, a parasitic tissue infestation caused by larval cysts (cysticercus cellulosae) of the pork tapeworm, *Taenia solium*, most commonly affects the central nervous system, subcutaneous tissue, skeletal muscle, heart muscle, and the eye. Ocular adnexal infestation in cysticercosis can involve various sites within the eye like vitreous humor, subretinal tissue, extraocular muscle, and lacrimal gland. In this case report, a lump slightly below and medial to inner corner of eye, surgically removed from a 21-year-old male patient, was diagnosed as case of lacrimal sac cysticercosis on histopathological examination. To our knowledge, this is the one of rare sites for manifestation of cysticercus cellulosae.

## 1. Introduction

*Cysticercosis* is a parasitic tissue infestation caused by larval cysts (cysticercus cellulosae) of the pork tapeworm,* Taenia solium*. It is acquired by ingestion of the infective cysticerci in undercooked pork or ingestion of eggs of* T. solium* in contaminated water, food, or vegetables and/or regurgitation of eggs from the small intestine [1, 2]. Cysticercosis occurs globally, but the highest cases are reported from Latin America, Asia, and Africa [3].

The sites for predilection for development of cysticerci are the central nervous system, subcutaneous tissue, skeletal muscle, cardiac muscle, and the eye [2]. The most common presentation is neurocysticercosis, parasitic manifestation of central nervous system. Ocular and periocular cysticercosis is less frequent, accounting for about 20% of cases. In the eye, the larval cysts lodge primarily in the vitreous humor, subretinal tissue, and the anterior chamber of the eye [3]. The extraocular muscle involvement is the commonest type of orbital and adnexal cysticercosis. Intraorbital cysticercosis can lead to blindness, if untreated. Association of orbital cysticercosis with systemic cysticercosis is quite rare [4]. Diagnosis of ocular involvement is facilitated by ultrasonography and computed tomography (CT) scan [5]. Case Presentation

### 1.1. Materials and Methods

**Figure 1:**
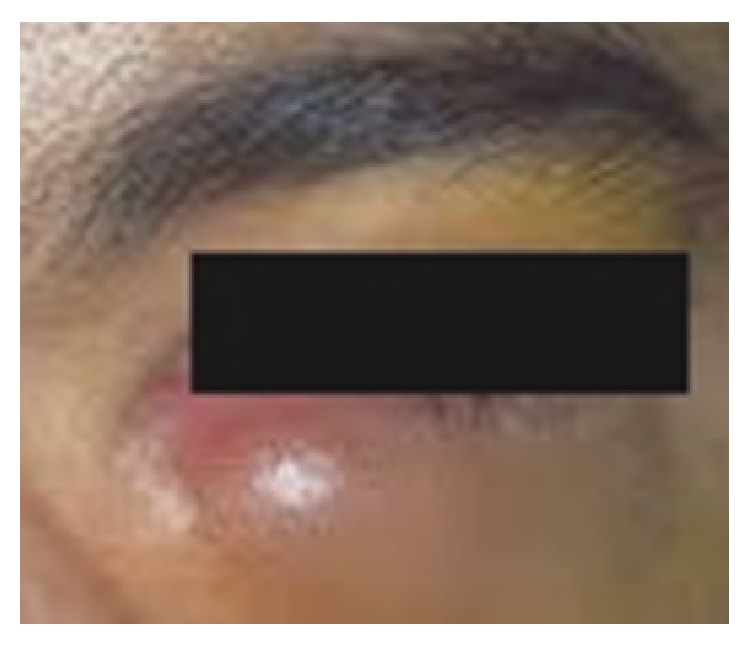
Swelling near the medial canthus of left eye.

An unusual manifestation of ocular cysticercosis was seen in 21-year-old male patient who presented with swelling located slightly below and medial to inner corner of left eye for about 1–1.5 months (Figure 1). He also complained of pain, redness, and discomfort in left eye. No other associated signs or symptoms like weakness of the ocular muscles, restriction of eye movement, or proptosis were seen. Bilateral pupils were normal in size and reacting to light. Fundus examination was normal. Visual acuity was 6/6 (Snellen chart). An excision biopsy was performed. The follow-up of patient was uneventful.

**Figure 2:**
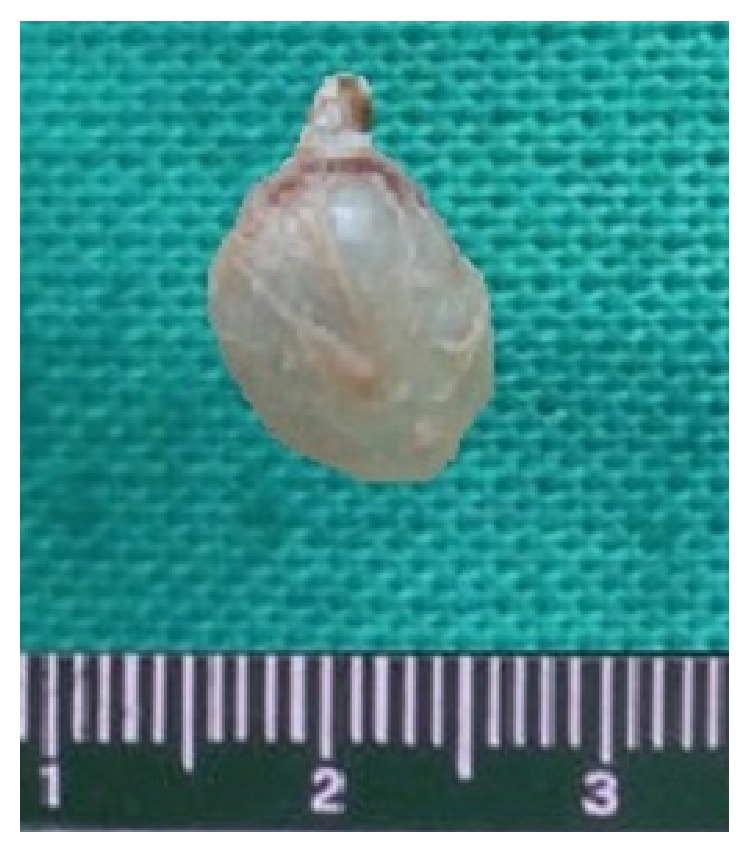
Gross morphology: cystic mass.

**Figure 3:**
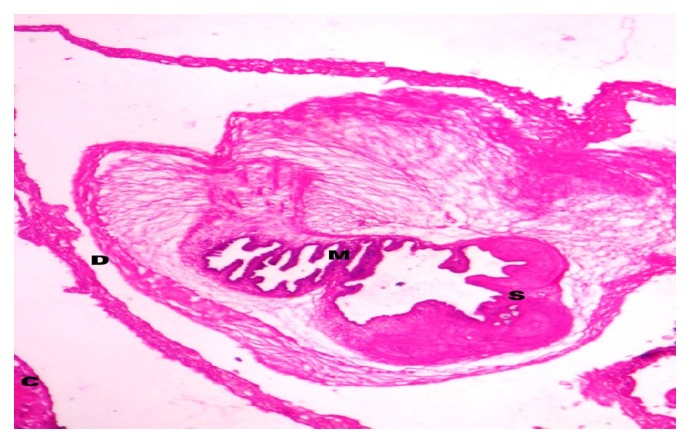
Histopathology: the connective tissue capsule (C) and larva within the cyst cavity surrounded by the double layered membrane (D). The duct-like invagination, lined by the homogeneous membrane (M) and scolex (S) (haematoxylin and eosin ×10).

**Figure 4:**
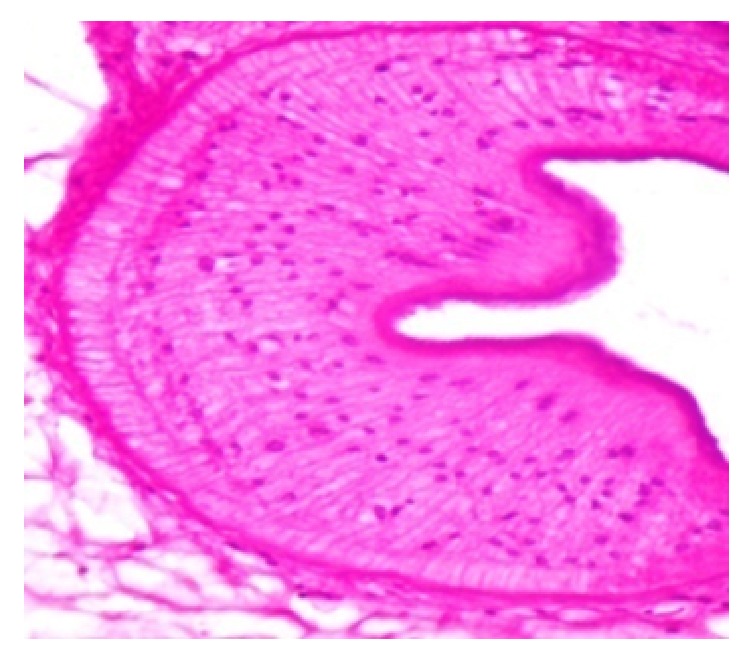
High power view of scolex (haematoxylin and eosin ×100).

The gross specimen was a globular cystic mass (1.6 × 1.2 × 1.3 cm) with slimy material (Figure 2). Cut section showed a cystic cavity with a white dot and scanty watery clear fluid. The histopathological features confirmed the diagnosis of cysticercosis. Histological examination revealed a cystic cavity, surrounded by a capsule of fibrous connective tissue with minimal inflammatory reaction and the cysticercus cellulosae. Worm morphology showed thick cyst wall thrown into duct-like invagination, lined by homogeneous membrane along with the scolex (Figures 3 and 4).

## 2. Discussion

Human cysticercosis is a parasitic infestation caused by encystment of the larvae (cysticercus cellulosae) of the pork tapeworm,* Taenia solium* [6]. Man is the intermediate host in the life cycle of* Taenia solium* who acquires infection by consuming food contaminated by eggs and can harbor several hundred cysticerci in various tissues and organs. Severity and symptomatology of cysticercosis vary according to the localization of the parasite, number of cysticerci, their stage of development (young, mature, intact, and degenerate), morphology (vesicular or racemose), location in the central nervous system, and the reaction of the host [3].

Neurocysticercosis is the most common form of systemic involvement [7]. Ocular and adnexal cysticercosis represents 13% to 46% of systemic disease. Ocular cysticercosis can involve any part of the eye. In the eye, the most common site for cysticercus cellulosae is vitreous and subretinal spaces and the anterior chamber of the eye [3], followed by orbit and adnexal tissues. Approximately 4% involve the eyelid or orbit, 20% involve the subconjunctival space, 8% involve the anterior segment, and 68% involve the posterior segment (subretinal and intravitreal) [8]. The extraocular muscle is the commonest type of orbital and adnexal cysticercosis [4, 8, 9]. An Indian study has reported ocular involvement in 12.8% of all cases of cysticercosis with the majority located in the subconjunctival space and only 1 case showing orbital involvement [10]. Another study from India has also reported subconjunctival cysticerci as the most common site of ocular cysticercosis (60%) [11].

According to a recent study, the most common site for cyst was the anterior orbit (69%), subconjunctival space (24.6%), posterior orbit (5.8%), and the eyelid (0.6%) [12]. The posterior segment is more commonly affected in western countries, whereas in India the cysts are more often subconjunctival [13]. Lacrimal gland involvement may cause a chronic dacryoadenitis and enlargement of the gland [14]. Imaging modalities like ultrasonography and computed tomography are commonly used for evaluation of orbital cysticercosis. The patients can be treated with systemic steroids and albendazole; however, surgical excision is the treatment of choice.

In the developing countries, orbital cysticercosis can have myriads of clinical presentation depending on the site of lodgment. In review of the literature from India, intraorbital cysticercosis involving orbital muscle [15], optic nerve [16], and 6 cases of adnexal cysticercosis [17] have been reported. Although the exact incidence is still unknown, lacrimal sac cysticercosis is very uncommon presentation and only few cases have been reported from India including this case [18, 19]. Thus a high degree of clinical suspicion and improved knowledge of the typical and atypical presentations of orbital cysticercosis can help in clinching the correct diagnosis.
